# Study on the Compressive and Tensile Properties of Latex-Modified Cement Stone

**DOI:** 10.3390/ma17194868

**Published:** 2024-10-03

**Authors:** Lianzhi Yang, Jie Zhang, Jiyun Shen, Hongfei Ji

**Affiliations:** 1 School of Civil and Resource Engineering, University of Science and Technology Beijing, Beijing 100083, China; m202210043@xs.ustb.edu.cn; 2 CNPC Engineering Technology R&D Company Limited, Beijing 102206, China

**Keywords:** latex-modified cement stone, compressive strength, tensile strength, strength criterion parameters

## Abstract

The integrity of wellbores is essential for the safe and efficient operation of drilling activities. Cement plays a critical role in this process, serving as a primary barrier that isolates the casing from the surrounding formation. To ensure the proper application of cement in wells, a thorough understanding of its mechanical properties is essential. Latex-modified cement stone (LMCS) offers significant advantages due to its anti-channeling, anti-corrosion, and mechanical characteristics. This study examined the mechanical properties of LMCS through uniaxial and triaxial compression and Brazilian splitting tests. Under uniaxial compression, the elastic modulus, Poisson’s ratio, and compressive strength of LMCS were found to range from 4.08 to 8.29 GPa, 0.05 to 0.46, and 15.82 to 22.21 MPa, respectively. In triaxial compression tests with confining pressures of 2 MPa, 4 MPa, 6 MPa, 8 MPa, and 10 MPa, the elastic modulus ranged from 4.48 to 6.87 GPa, Poisson’s ratio from 0.05 to 0.16, and compressive strength from 27.38 to 39.58 MPa. The tensile strength of LMCS ranged from 2.34 to 3.72 MPa. Moreover, the compressive strength of LMCS increased with confining pressure, showing enhanced resistance to failure due to the confining effect. However, the rate of increase gradually diminished. Strength criteria for LMCS, including Mohr–Coulomb and Drucker–Prager parameters, were derived from the triaxial compression tests. These strength criteria parameters provide a useful reference for developing the constitutive model of LMCS and for simulating triaxial compression conditions. The findings of this research offer valuable insights that can guide the construction of oil and gas wells.

## 1. Introduction

As the global energy demand continues to rise, the need for geological storage of renewable energy in new oil and gas wells has become increasingly significant. Oil well cement paste remains the most widely used material for zonal isolation in wells, whether for hydrocarbon or geothermal energy recovery, or for geological carbon dioxide sequestration [1]. Cement plays a critical role as a barrier, ensuring pressure isolation between the formation and casing in wellbores [2,3]. The cement ring bonds the casing to the rock formation, creating a cohesive unit that is capable of withstanding forces from the surrounding formations and changes within the wellbore [4,5,6,7]. Unlike conventional Portland cement, which is used in general construction, wellbore cement must perform in high-temperature, high-pressure, and sometimes corrosive environments, such as acidic media like CO_2_ [8,9]. Many researchers have studied the mechanical and rheological properties of traditional and modified cements [10,11]. However, in oil and gas well construction, traditional and partially modified cements have been found to be inadequate [12]. Latex-modified cement stone (LMCS) has shown notable advantages, including preventing seepage, resisting corrosion, and offering superior mechanical performance. The compressive strength and elastic modulus of LMCS make it especially well suited for oil and gas wells [3,13], leading to its widespread adoption both domestically and internationally [14,15,16,17]. Further research on the mechanical properties of LMCS can provide essential theoretical and technical support for its effective application in wellbores.

Understanding the tensile and compressive characteristics of cement stone through experimental studies is crucial for analyzing its failure process and holds significant implications for engineering applications. Uniaxial and triaxial tests are commonly used to determine the compressive properties of cement stone and obtain parameters such as its compressive strength, elastic modulus, and Poisson’s ratio. Liu et al. [18] conducted uniaxial and triaxial compression tests on the cementitious material of an oil well, and they established a nonlinear deformation model for the material. Su et al. [19] performed triaxial compression tests and revealed the noticeable plastic behavior of cementitious materials under high confining pressures. Mahmoud et al. [20] cured a G-grade cement paste at different temperatures and conducted uniaxial compressive strength tests on the specimens. They observed a significant reduction in the compressive strength of the different formulations of oil well cement pastes under a high-temperature (300 °C) curing environment. Lima et al. [21] conducted quantitative uniaxial and triaxial compression tests on saturated mortar and dry mortar. The results showed that the mortar’s peak strength decreased with increased saturation. Aside from the compressive properties of cement stone, its tensile strength characteristics have also been widely investigated, with Brazilian splitting tests commonly used to determine these tensile properties and obtain parameters such as tensile strength. Quercia et al. [22] explored the relationship between the tensile strength of cement mortar and its micro-fiber content using Brazilian splitting tests. Zhang et al. [23] investigated the tensile strength of cementitious materials at different scales through Brazilian splitting tests and numerical simulations. Sun et al. [24] conducted Brazilian splitting tests on 3D-printed cement-based materials at different temperatures and strain rates, revealing an increase in the tensile strength of cement-based materials with higher temperatures and lower strain rates. In addition to conventional tensile and compressive measurements, some scholars have examined the compressive and tensile properties of materials under cyclic loading. Xu et al. [25] studied the stress–strain behavior and damage evolution of *Φ*50 mm × h98 mm short columns made of rubber cement mortar; the study was conducted under 10 cycles of uniaxial compression. Zhang et al. [26] explored the mechanical characteristics and failure mechanisms of cement mortar through triaxial compression tests and cyclic loading–unloading experiments. It can be seen from the presented literature review that there are relatively few studies that have involved systematically studying the tensile and compressive performance of LMCS. Furthermore, the process of obtaining different strength criteria parameters for LMCS to study its constitutive relationships remains unclear. This demands further research on the mechanical properties of LMCS to provide a theoretical reference and technical support for the construction of oil and gas wells.

The LMCS used in this study is an oil and gas well cement that is already utilized in the industry. To determine the crucial mechanical parameters of LMCS, including its compression and tensile properties, in this study, we conducted comprehensive mechanical tests, including uniaxial compression, triaxial compression, and Brazilian splitting tests, on LMCS. The elastic modulus, Poisson’s ratio, compressive strength, and tensile strength of LMCS were measured and analyzed. Additionally, the Mohr–Coulomb (M-C) and Drucker–Prager (D-P) strength criteria parameters were derived from the triaxial compression tests. These findings not only provide crucial parameters for investigating the constitutive relationship and simulating the triaxial behavior of LMCS, but also offer a scientific foundation for its application in cementing engineering practices.

## 2. Experimental Materials and Procedures

### 2.1. Sample Preparation

The latex-modified cement slurry (LMCS) formulation, detailed in Table 1, includes cement, water, latex, stabilizer, and two types of defoamers: latex defoamer and general defoamer. Latex reduces the elastic modulus of the cement stone, thereby extending the lifespan of the cement sheath in oil and gas wells [27,28]. Stabilizers enhance the stability of the cement slurry system, ensuring uniform consistency [29]. Defoamers minimize bubbles in the slurry, which in turn increases the strength of the cement stone. Compared to conventional cement stone, LMCS exhibits higher compressive strength and a lower elastic modulus.

**Figure 1:**
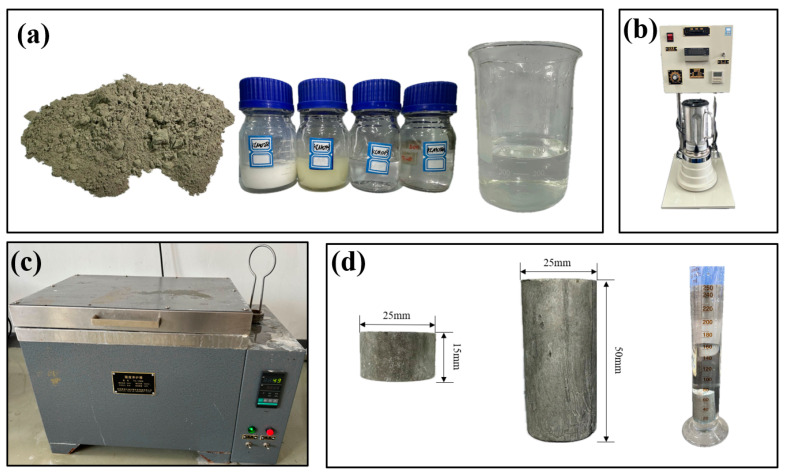
Manufacturing process of the LMCS specimens: (**a**) cement, admixture, and water; (**b**) constant-speed mixer; (**c**) curing box; and (**d**) Brazilian splitting specimen, compression specimen, and drainage method for density measurement.

A mixture of cement and water with a mass ratio of 1.0:0.32 was used to create the cylindrical specimens (Figure 1a). The cement mortar was prepared using a constant-speed mixer in accordance with the GB/T 19139-2012 standard [30] (Figure 1b). First, butter was evenly applied to the inner surface of a cubic mold with a cylindrical indentation (25 mm in diameter and 50 mm in height). The thoroughly mixed cement mortar was then transferred into the mold, and any air bubbles were carefully removed with a glass rod. The layered cement specimens were placed in a curing box under controlled conditions—a temperature of 50 ± 5 °C and humidity of 95% ± 5% (Figure 1c). After seven days of curing, the mold was removed. The ends of the specimens were trimmed to ensure smoothness, resulting in final specimens with a diameter of 25 mm and a height of 15 mm, which were prepared for Brazilian splitting tests (Figure 1d). This study focuses on examining the mechanical properties of LMCS after seven days of curing. The cement stone has a density of 1.88 g/cm³. The porosity and pore volume were determined using the drainage method, with the porosity calculated by measuring the sample’s mass in both saturated and completely dry states to derive the pore volume. As shown in Table 2, the average porosity of LMCS is 10.70%.

In this study, each specimen received a systematic label following a specific format: “D-number”, “S-number”, or “V-number”, where “D” represents specimens designated for the uniaxial compression test, “S” for the triaxial compression test, and “V” for the Brazilian splitting test. For example, a specimen labeled “S-2” signifies the second specimen intended for a triaxial compression test.

### 2.2. Experimental Procedure

**Figure 2:**
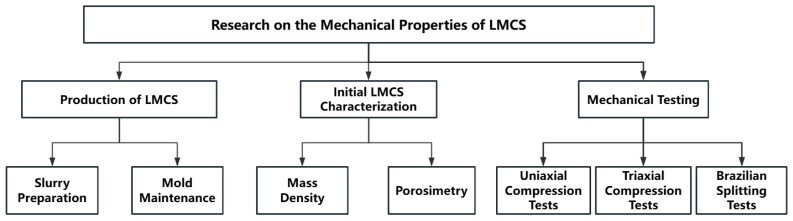
Experimental program summarized in flowchart.

The experimental characterization and testing of LMCS include preparation and curing, mass density measurement, porosity measurement, compressive strength measurement, compressive strength measurement under different confining pressures, and Brazilian splitting test for measuring tensile strength. An overview of the test program is provided in Figure 2. All mechanical tests in this study were conducted following the SY/T 6466-2016 standard [31].

**Figure 3:**
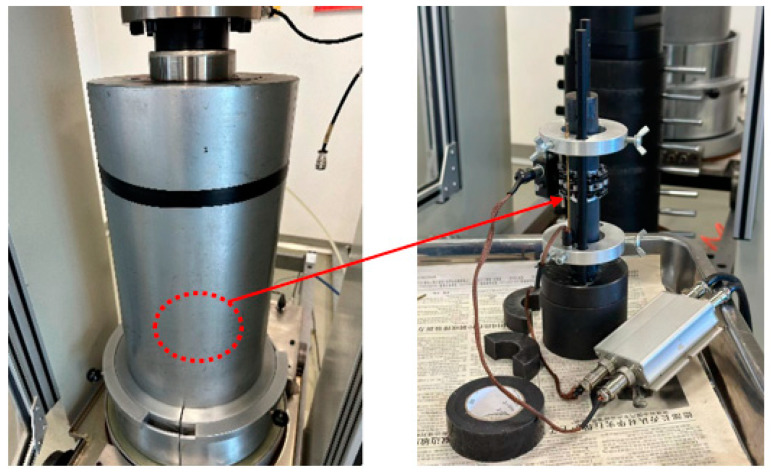
TAW-1000 system used to perform the triaxial compression test.

The TAW-1000 electro-hydraulic servo cement stone testing system was used to conduct the uniaxial, triaxial, and Brazilian splitting experiments. This system is operated by two high-precision pumps that produce axial and confining pressures, supporting maximum loads of 1000 kN and 150 MPa, respectively. The triaxial compression test, as shown in Figure 3, obtained accurate measurements of axial and radial strain using a spring-loaded extensometer, and the entire testing process was managed using a dedicated computer. The sample was placed in the middle of the extensometer and the initial deformation was adjusted to a display range of ±500 μm. The position of the intermediate sample was adjusted and confining pressures of 2 MPa, 4 MPa, 6 MPa, 8 MPa, and 10 MPa were applied during five sets of experiments. An axial displacement control mode was employed during testing, and a consistent displacement rate of 0.05 mm/min was maintained until the specimen fractured. Throughout the experiment, data sampling occurred every 0.1 s.

**Figure 4:**
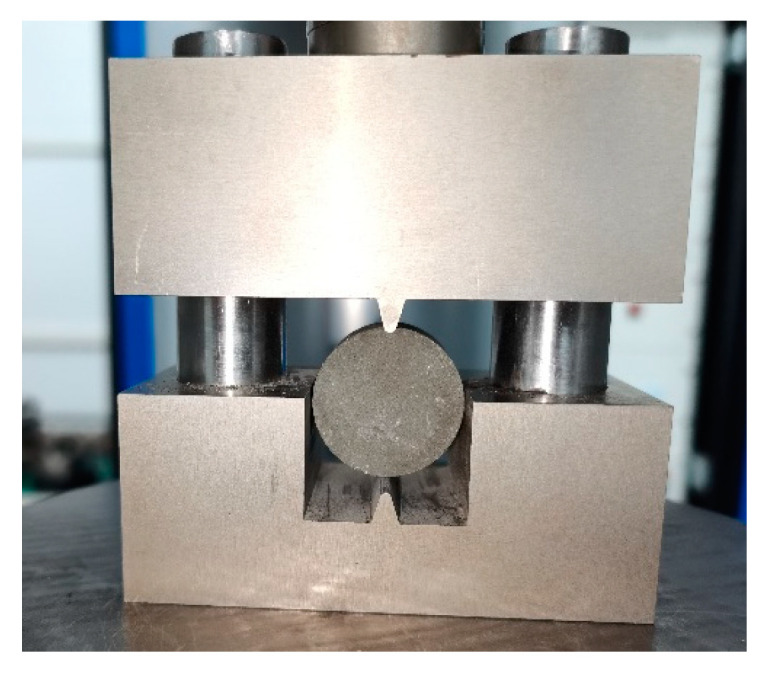
TAW-1000 system used to perform the Brazilian splitting test.

The Brazilian splitting technique was employed to evaluate the tensile strength of cement stone specimens through disc splitting tests within the testing system, as illustrated in Figure 4. For this experiment, the indenter loading method was chosen due to its minimal test variability and consistent observation of failure patterns. A connecting rod was affixed to a universal testing machine and connected to the Brazilian splitting apparatus. The specimen was centrally positioned within the grip, ensuring a careful alignment to facilitate a fracture at the midpoint of the disc specimen.

## 3. Experimental Results and Discussion

### 3.1. Study on Uniaxial Compression Properties

During the uniaxial compression test, axial displacement control was adopted in the loading process with a loading rate of 0.05 mm/s. Loading continued after the sample reached peak stress in order to obtain a complete stress–strain curve. Six uniaxial compression tests, labeled D-1 through D-6, were conducted on different samples.

**Figure 5:**
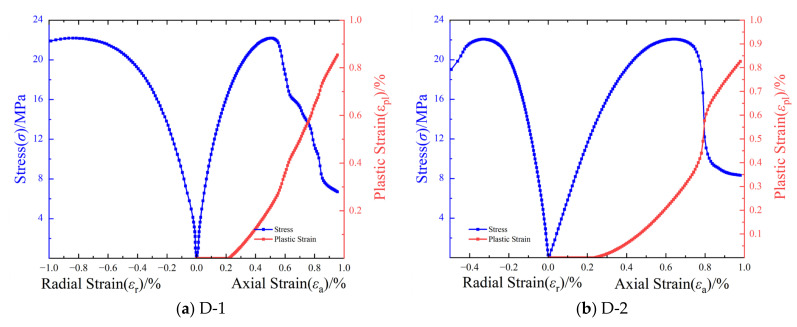

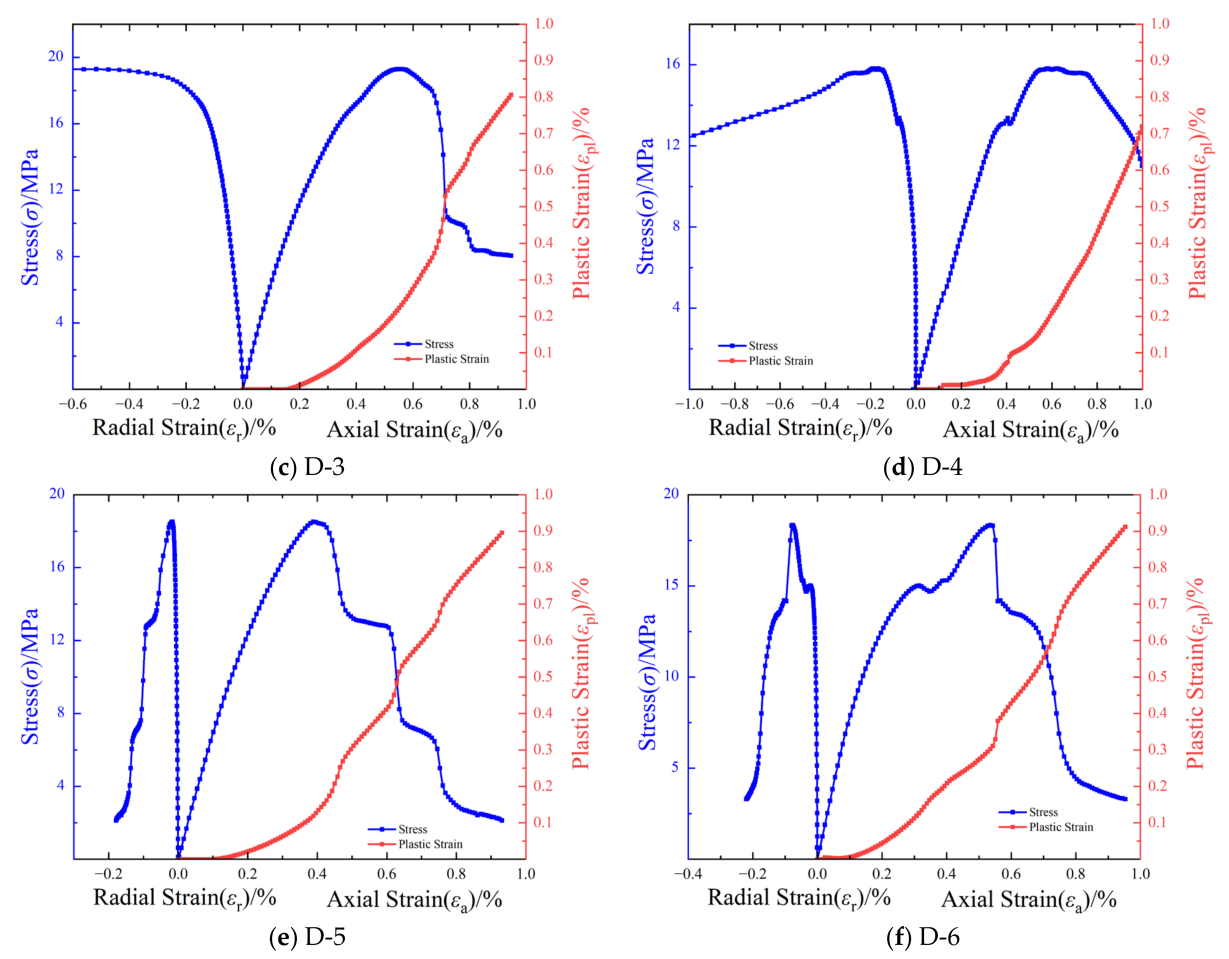
Stress–strain curves and plastic strain curves of LMCS under uniaxial compression: (**a**) D-1; (**b**) D-2; (**c**) D-3; (**d**) D-4; (**e**) D-5; (**f**) D-6.

Figure 5 illustrates the stress–strain curves and plastic strain curves of LMCS under uniaxial compression. First, the stress–strain curve, representing the compaction stage, exhibits a slightly upward, curved, and concave profile, which is indicative of microcracks compressing and closing. Due to the uneven distribution of pores in the cement matrix, the duration of these processes varies. As the load increases, the specimen shifts to a linear elastic compression stage, which is characterized by a directly proportional stress–strain relationship. Upon reaching the crack propagation stage, which is marked by the evolution of microcracks into macroscopic cracks and a deviation from the linear path, the specimen fails at the curve’s peak, with the post-peak stress rapidly decreasing. Notably, the Poisson’s ratio of specimen D-1 exceeds 1, which is potentially the result of improper operation of the radial extensometer. Therefore, D-1 is not considered when discussing the range of the LMCS Poisson’s ratio. During the crack propagation stage, specimens D-4 and D-6 exhibit significant yield points that are not observed in other specimens. In addition, the residual strength of specimens D-1, D-2, and D-3 at an axial strain of 1.0% is approximately 8 MPa, while specimens D-5 and D-6 exhibit a residual strength of approximately 3 MPa. The experimental results indicate that the strength of the D-4 sample is somewhat lower compared to that of other samples. This discrepancy is attributed to the heterogeneity of the cement paste. Specifically, the cement slurry poured into the D-4 mold contained higher water content and more air bubbles, resulting in reduced strength. This was elucidated in the original text. This is consistent with previous research findings [12,32]. The uniaxial compression tests revealed a sharp decrease in stress post-peak, highlighting the brittleness of LMCS under uniaxial compression.

The plastic strain is obtained through the stress–strain curve. The calculation equation is shown as follows [33,34]:

(1) $$\varepsilon_{pl}=\ln(1+\varepsilon_{norm})-\frac{\sigma_{norm}(1+\varepsilon_{norm})}{E}$$

where $\varepsilon_{norm}$ is the nominal strain and $\sigma_{norm}$ is the nominal stress.

Figure 5 shows that before entering the crack propagation stage, LMCS undergoes elastic deformation with zero plastic strain. As the crack propagation stage is reached, a gradual increase occurs in the plastic strain. Upon entering the post-peak stage, the plastic strain curve rises sharply, accompanied by a significant decrease in stress. The plastic strain curve transitions from a downward depression to an upward protrusion. Notably, the plastic strain of LMCS starts to rise rapidly at approximately 0.2–0.4%, indicating failure under uniaxial compression conditions when the cumulative plastic strain accumulates to this range. Additionally, the slope of the plastic strain curve increases as the slope of the stress–strain curve decreases, and the two trends are inversely related. Importing stress and plastic strain into numerical simulation software allows for effective simulation of the hardening and softening stages, facilitating numerical simulations of the uniaxial compression of LMCS.

The elastic modulus is calculated using the secant method [35,36]. In this method, the elastic modulus is defined as the tangent slope of the stress–strain curve. Given the brevity of the compaction stage and the axial strain settling at less than 0.01%, this phase is excluded from the elastic modulus calculation. As the axial strain at the end of the elastic stage in Figure 4 typically approaches 0.1%, the elastic modulus is calculated in this study using 0% and 0.1% as the starting and ending points of the axial strain during the loading phase. The calculation equation is as follows:

(2) $$E=\frac{\sigma_{a_{0.1}}}{\varepsilon_{a_{0.1}}}$$

where $\varepsilon_{a_{0.1}}$ is obtained from the axial strain.

Poisson’s ratio (ν) is defined as the ratio of radial strain ($\varepsilon_{r}$) to axial strain ($\varepsilon_{a}$) [18]:

(3) $$\upsilon =-\frac{\varepsilon_{r}}{\varepsilon_{a}}=-\frac{\sigma_{a_{0.1}}/\varepsilon_{a_{0.1}}}{\sigma_{a_{0.1}}/\varepsilon_{r_{0.1}}}$$

where $\varepsilon_{r}$ and $\varepsilon_{a}$ are radial strain and axial strain, respectively The terms $\sigma_{a_{0.1}}$ and $\varepsilon_{r_{0.1}}$ are the corresponding stress and radial strain when the axial strain is $\varepsilon_{a_{0.1}}$. The minus sign in the Poisson’s ratio formula is required because the two strain values in Equation (3) have opposite signs.

The results of the uniaxial compression test are presented in Table 3. The elastic compression modulus ranges from 4.08 to 8.29 GPa, with an average of 6.47 GPa. Poisson’s ratio ranges from 0.05 to 0.46, with an average of 0.20. The compressive strength ranges from 15.82 to 22.21 MPa, averaging 19.38 MPa. The compression test results showed a significant change in Poisson’s ratio. The reason for this is that cement stone is a heterogeneous material. The deformation of cement stone in different directions during compression is influenced by the concentration of cement in that direction and the number of bubbles present. This is consistent with previous research findings [37].

The compressive strength and elastic modulus of traditional cements or other modified cements used in oil and gas well construction are shown in Table 4 [18,21,32,38]. The compressive strength and elastic modulus of LMCS in Table 3 are relatively low in comparison. However, compressive strength that is slightly higher than the formation confining pressure and a lower elastic modulus are more suitable for application in oil and gas wells [13].

**Figure 6:**
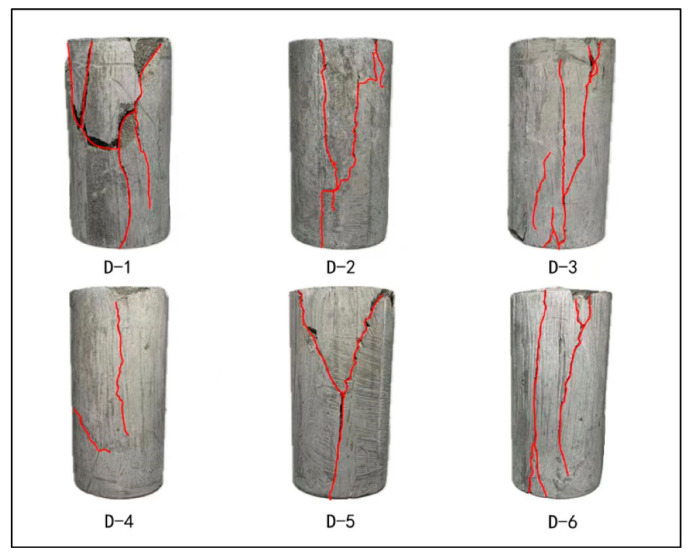
Cement stone specimens after uniaxial compression (the red lines represent cracks).

Figure 6 illustrates the post-uniaxial compression condition of each cement stone specimen. The failure of the sample was sudden and loud, with most specimens developing cracks throughout. Notably, D-4 did not form such a crack, and it exhibited a significantly lower compressive strength compared to others. Therefore, D-4 was excluded from the subsequent discussion on the crack morphology in LMCS. Specimens D-1, D-2, D-3, and D-5 showed a distinct Y-shaped crack pattern, while D-6 developed two vertical cracks.

### 3.2. Study on Triaxial Compression Properties

During the triaxial compression test, the loading was performed using axial displacement control with a loading rate of 0.05 mm/s. The procedure involved gradually applying the confining pressure to a predetermined value and allowing it to stabilize before initiating axial loading. After the specimen attained peak stress, loading was sustained for a specific period of time to acquire a complete stress–strain curve. The five different samples, labeled S-1 to S-5, underwent triaxial compression testing under confining pressures of 2 MPa, 4 MPa, 6 MPa, 8 MPa, and 10 MPa, respectively.

**Figure 7:**
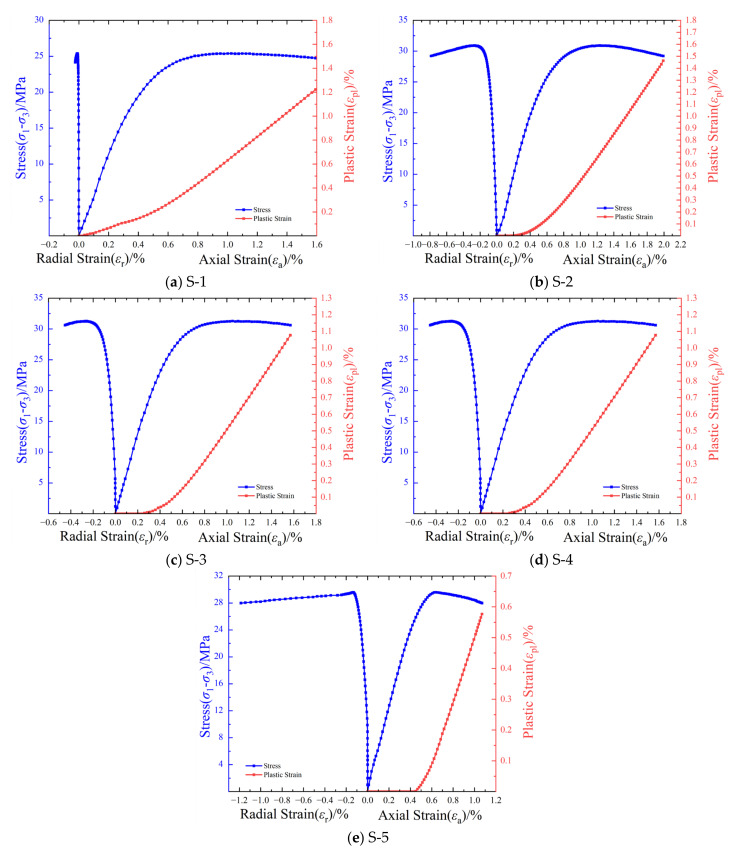
Stress–strain curves and plastic strain curves of saturated LMCS under triaxial compression: (**a**) S-1; (**b**) S-2; (**c**) S-3; (**d**) S-4; (**e**) S-5.

Figure 7 illustrates the stress–strain curves and cumulative plastic strain curves of LMCS under triaxial compression. During the initial loading stage and linear elastic compression stage, the stress–strain curves of the triaxial compression specimen are similar to those of the uniaxial compression specimen. However, unlike the uniaxial compression specimens, peak strain softening is more stable during the yield and post-peak stages. Upon reaching peak stress, the triaxial compression specimen enters the plateau period, exhibiting a material performance closer to that of ductile materials. After the linear elastic stage, the plastic strain of the specimen increases continuously, with the plastic strain curve consistently showing a slightly downward concave shape. The initiation of plastic strain in LMCS occurs later and later as the confining pressure increases. The trend in the plastic strain curve differs from that under uniaxial compression, and the slope of the plastic strain remains stable during the post-peak stage. The stress–radial strain curve exhibits a similar shape to that of the stress-axial strain curve.

The results of the triaxial compression tests of the cement stone specimens are presented in Table 5. The data show that the elastic compression modulus of the specimens ranged from 4.48 to 6.87 GPa, with an average of 5.86 GPa. The Poisson’s ratio varied from 0.05 to 0.16, with a mean value of 0.11. Regarding compressive strength, the specimens exhibited a range of 27.38 to 39.58 MPa, with an average strength of 35.60 MPa. As the confining pressure increases, the compressive strength of the specimen gradually increases.

The M-C strength criterion is widely recognized and utilized in the field of geotechnical engineering [39,40,41]. This study adopts the M-C criterion as the defining measure for LMCS failure. Mohr circles, originating from triaxial test results, visually depict the stress condition at any given point, illustrating both normal and shear stresses. These circles plot normal stresses (*σ*_1_ and *σ*_3_) on the horizontal axis, with a range of Mohr circles reflecting different confining pressures (*σ*_3_) and associated vertical stresses (*σ*_1_). On planes where shear stresses vanish, principal stresses emerge, correlating with the horizontal axis. At this point, *σ*_1_ and *σ*_3_ are recognized as principal stresses. To ascertain shear strength parameters, tangents are drawn to the series of Mohr circles for the specimens. The intersection of a tangent with the vertical axis determines the specimen’s cohesion (*c*), and the tangent’s angle of inclination reveals the internal friction angle (*φ*). The distinction in principal stresses (*σ*_1_–*σ*_3_) is represented by the diameter of the Mohr circle. Following the approach outlined by Das [42], tangents are drawn to the Mohr circles. The M-C failure criterion, reformulated to highlight failure stresses, is articulated as follows:

(4) $$\sigma_{1}=\sigma_{3}\;\tan^{2}(45^{\circ }+\frac{\varphi }{2})+2c\;\tan(45^{\circ }+\frac{\varphi }{2})$$

where c = cohesion and φ = the angle of internal friction.

Cohesion and the angle of internal friction, which are fundamental characteristics of materials, play a crucial role in shear strength calculation. For each series, shear strength parameters were determined, enabling the formulation of the M-C failure envelope equation. Applying the known values of cohesion and the angle of internal friction, which were derived from the Mohr circle corresponding to a particular stress condition, allowed the shear strength to be calculated based on the provided equation.

(5) τ=c+σtanφ

**Figure 8:**
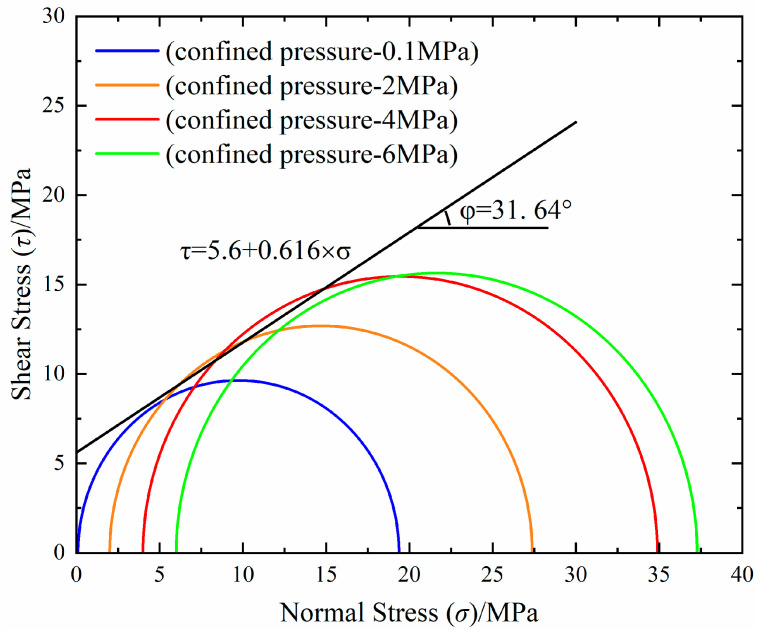
M-C failure envelope for latex-modified cement.

The values of c and φ in Equation (5) are obtained by fitting the envelope lines using triaxial compression test data under different confining pressures combined with Equation (4). The intercept of the outer tangent on the vertical axis (shear stress *τ*) represents the generalized cohesion, while the angle between the outer tangent and the horizontal axis (axial stress *σ*_1_) indicates the generalized internal friction angle *φ*. Figure 8 illustrates the M-C failure envelopes of LMCS. The image displays the cohesive force and internal friction angle of LMCS as follows: c = 5.6 MPa; φ = 31.64°.

The D-P strength criterion is also a common geotechnical strength theory. The D-P yield criterion [43,44] is expressed as follows:

(6) $$F(I_{1},J_{2})=\alpha I_{1}+\sqrt{J_{2}}-k=0$$

(7) $$I_{1}=\sigma_{xx}+\sigma_{yy}+\sigma_{zz}$$

(8) $$J_{2}=\frac{1}{2}[{\left(\sigma_{xx}-\sigma_{yy}\right)}^{2}+{\left(\sigma_{yy}-\sigma_{zz}\right)}^{2}+{\left(\sigma_{zz}-\sigma_{xx}\right)}^{2}]+3(\sigma_{xy}^{2}+\sigma_{yz}^{2}+\sigma_{zx}^{2})$$

where *I*_1_ and *J*_2_ are the first invariants of the stress tensor and the second invariant of the stress deviator tensor, respectively. The terms *α* and *k* are the frictional parameter and hardening function, respectively. Under the plane strain state, *α* and *k* can be calculated based on the M-C criterion parameters as follows [45]:

(9) $$\alpha =\sin\varphi /[\sqrt{3}\sqrt{3+\sin^{2}\varphi }]=0.17$$

(10) $$k=3c\cos\varphi /[\sqrt{3}\sqrt{3+\sin^{2}\varphi }]=4.09$$

The obtained results can provide parameters for simulating the triaxial compression of LMCS using different strength criteria.

**Figure 9:**
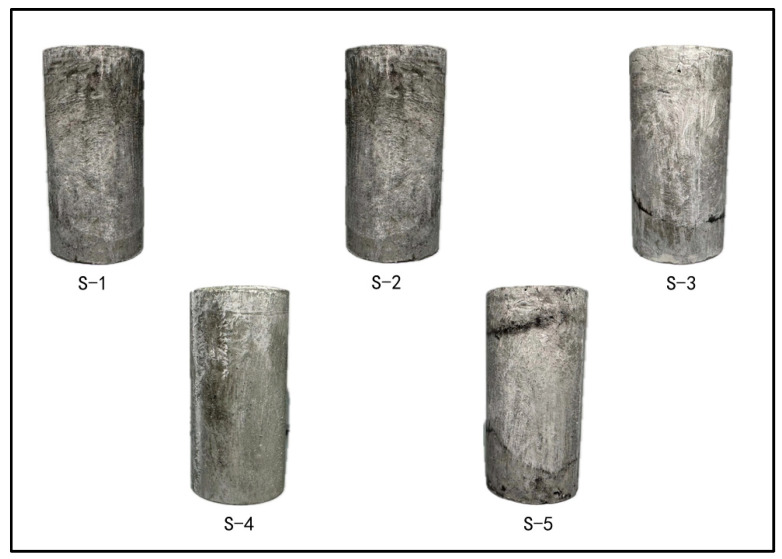
Cement stone specimens after triaxial compression.

Figure 9 displays the post-triaxial compression state of the cement stone specimens. Notably, the samples did not develop any macroscopic cracks. An analysis of the stress–strain curves under triaxial compression reveals that LMCS typically does not form macroscopic cracks when exhibiting toughness. The reason for this result is that the toughness exhibited by LMCS under triaxial compression slows down the propagation of microcracks and delays their failure [46].

**Figure 10:**
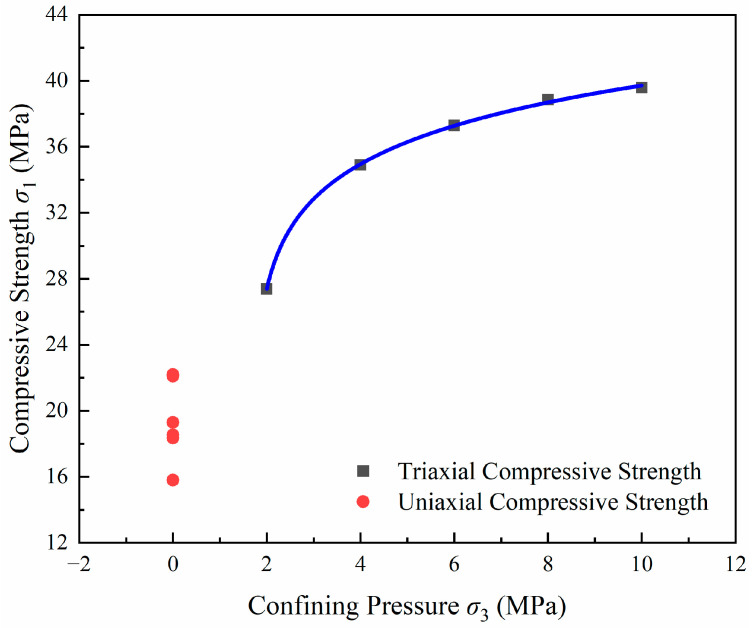
Relationship between compressive strength and confining pressure of specimens.

Figure 10 shows that the triaxial compressive strength of LMCS is significantly higher than the uniaxial compressive strength. Observations from the triaxial compression test results of LMCS reveal that the compressive strength increases with the confining pressure, but as the confining pressure gradually increases, the rate of increase in compressive strength gradually decreases. Based on these results, it can be speculated that the strength of LMCS does not continue to increase significantly under high confining pressures. This phenomenon has also been observed in previous research [47].

### 3.3. Study on Tensile Properties

During the Brazilian splitting tests, control over axial displacement was maintained throughout the loading phase, with a set loading rate of 0.05 mm/s, continuing until sample failure. These tests, identified as V-1 to V-6, were performed on various samples.

**Figure 11:**
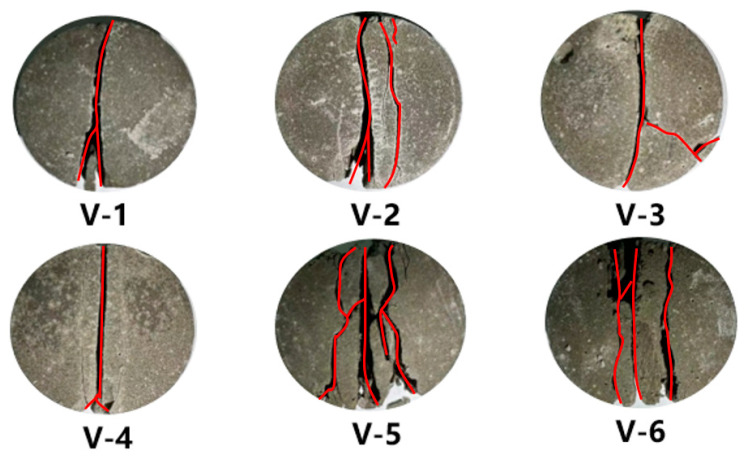
Cement stone specimens after splitting (the red lines represent cracks).

Figure 11 illustrates the fracture patterns observed in LMCS during the Brazilian tests. The cracks were initiated at the center of the cement stone specimens, and they quickly propagated vertically, creating a through-and-through vertical fracture that bisected the specimen along the radial direction of the applied load. In most cases, a support crack formed in the lower part of the specimen. For samples V-2, V-5, and V-6, after the formation of the initial crack, additional cracks of the same type appeared in the vicinity of the original crack. These findings closely align with outcomes from prior Brazilian tests conducted on cement stone, demonstrating consistent fracturing behavior [24].

**Figure 12:**
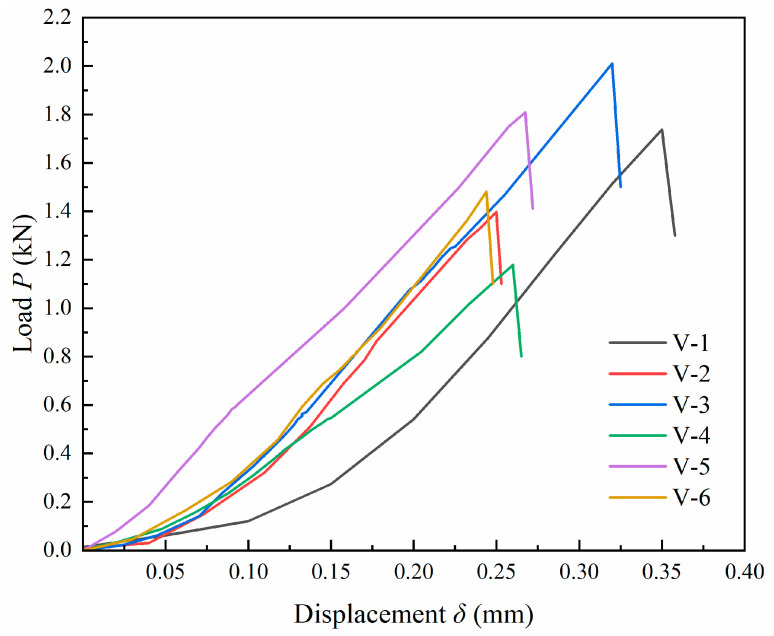
Load–displacement curves of Brazilian splitting test.

Figure 12 shows the load–displacement curve of LMCS specimens under the Brazilian splitting test. Initially, the curve exhibits a slightly upward concave shape. Before the peak strength is reached, the yielding phase is not apparent. After peak strength is achieved, the curve rapidly descends linearly, entering the failure phase, which corresponds to the fracture expansion state of disc specimens. Unlike the stress–strain curve under compression for LMCS specimens, the post-peak phase of the Brazilian splitting load–displacement curve is shorter, reflecting the distinct compression and tensile characteristics of LMCS. The ultimate failure load of LMCS specimens ranges from 1398 to 2011N, with an average value of 1602N.

In the Brazilian splitting tests, the formula below was used to calculate the tensile strength of the cement stone specimens after they reached their maximum load capacity:

(11) $$\sigma_{t}=\frac{2P_{\max}}{\pi DH}$$

where $\sigma_{t}$ is the tensile strength, $P_{\max}$ denotes the ultimate load, *D* signifies the diameter of the specimen, and H is the thickness of the specimen.

The results of the LMCS Brazilian splitting test are shown in Table 6. The ultimate failure load of LMCS specimens ranges from 1179 to 2011N. The tensile strength of LMCS was calculated according to formula 11, with a range from 2.34 to 3.72 MPa. The variation in tensile strength is mainly caused by factors such as the unevenness of the cement slurry. These factors can influence the distribution of microcracks and pore structures, leading to differences in the tensile strength of the samples.

### 3.4. Discussion

**Figure 13:**
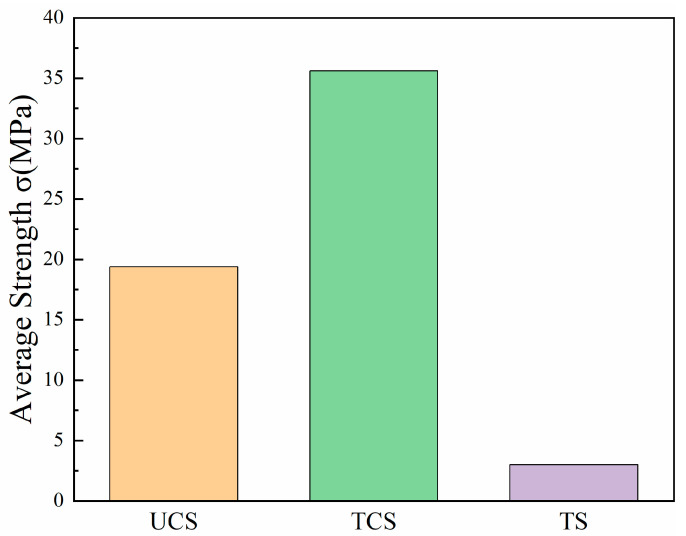
The average intensity of LMCS under different experimental conditions.

According to the SY/T6466-2016 Oil and Gas Well Cement Test Method [31], we have considered the uniaxial compression, triaxial compression, and tensile properties of LMCS. At a loading speed of 0.05 mm/min, the compressive strength, elastic modulus, Poisson’s ratio, and tensile strength of LMCS were obtained after curing at 50 °C and atmospheric pressure for 7 days. The comparison of strength under different experimental conditions of LMCS is shown in Figure 13. In the figure, UCS represents uniaxial compression strength, TCS represents triaxial compression strength, and TS represents tensile strength. When comparing the tensile strength with the compressive strength, the tensile compression ratio of LMCS is approximately 0.15. The tensile-to-compressive strength ratio of conventional cement typically ranges from 0.07 to 0.12 [48,49]. In the cementing process of oil and gas wells, the cement sheath must withstand a complex stress environment, including axial, radial, and shear stresses. The tensile strength of the cement sheath is crucial for preventing the initiation and propagation of microcracks. Conventional cement, as a result of its low tensile strength, is prone to forming microcracks under high-stress conditions, potentially leading to wellbore sealing failure. However, the higher tensile strength and tensile-to-compressive strength ratio of LMCS make it more suitable for scenarios requiring high strength and long-term sealing, such as deep wells or high-pressure wells. In particular, under triaxial stress conditions, LMCS’s higher strength enhances the stability of the cement sheath in complex stress environments, reducing the risk of wellbore leakage and casing failure. Therefore, LMCS is better suited for oil and gas well applications compared to conventional cement.

The experimental results presented in this article provide essential mechanical parameters for numerical calculations in failure analysis of oil and gas wellbore operations, thereby offering guidance for oil and gas well construction. However, all cement samples in this study were cured in a controlled environment at 50 °C under atmospheric pressure. The curing conditions were relatively uniform, and the number of test samples was relatively small. Future experiments are needed to explore how compressive strength in triaxial compression tests varies with temperature and confining pressure in order to better simulate actual downhole conditions.

## 4. Conclusions

This study aims to evaluate the mechanical properties of LMCS for oil and gas well construction. The samples were cured for 7 days at 50 °C and 95% humidity, and their mass density and porosity were measured. Uniaxial compression, triaxial compression, and Brazilian splitting tests were conducted on LMCS to determine its mechanical properties, including its compression and tensile properties. Calculations and analyses were performed to determine the elastic modulus of compression, Poisson’s ratio, and compressive and tensile strengths, considering the M-C and D-P strength criteria parameters of LMCS. The primary findings are summarized as follows:

(1) Under uniaxial compression, LMCS exhibited an elastic compression modulus ranging from 4.08 to 8.29 GPa, a Poisson’s ratio of 0.05–0.46, and compressive strength ranging from 15.82 to 22.21 MPa. The compressive strength and elastic modulus of LMCS are relatively low compared to traditional cement or other modified cement used in oil and gas well construction. Under triaxial compression, these values were 4.48–6.87 GPa for the modulus, 0.05–0.16 for Poisson’s ratio, and 27.38–39.58 MPa for compressive strength. The tensile strength ranged from 2.34 to 3.72 MPa, with a corresponding tensile–compression ratio of approximately 0.15.
(2) After uniaxial compression failure, LMCS often exhibited cracks, occasionally forming a Y-shaped failure path. Conversely, under triaxial compression, the specimens did not produce macroscopic cracks.
(3) The compressive strength of LMCS increased with the confining pressure. However, as the confining pressure continued to increase, the rate of increase in compressive strength gradually decreased.
(4) A failure envelope of the form was generated. The strength criterion parameters calculated through triaxial compression tests can provide a reference for studying the constitutive relationship of LMCS and simulating triaxial compression.
(5) All cement stone samples were cured in a controlled environment with a constant temperature of 50 °C. Further experiments are needed to discuss the variation in compressive strength with temperature and confining pressure in triaxial compression tests.
(6) The experimental results of this article can provide necessary mechanical parameters for numerical calculation of failure analysis of oil and gas wellbore operation, thereby guiding the construction of oil and gas wells.

## Figures and Tables

**Table 1:** Mix proportions of latex-modified cement.

Raw Material	Cement	Water	KCM028 (Latex)	KCM018A (Stabilizer)	KCM043 (Latex Defoamer)	KCM003 (Defoamer)
Proportion (%)	70.18	22.69	6.33	0.25	0.38	0.17

**Table 2:** Calculation of porosity in LMCS.

Specimen Number	Total Volume (mm^3^)	Pore Volume (mm^3^)	Porosity (%)
1	24,426.79	2740.00	11.22
2	23,910.93	2560.00	10.71
3	25,060.19	2370.00	10.17

**Table 3:** Uniaxial compression test results of cement stone specimens.

Specimen Number	Length *L* (mm)	Diameter *D* (mm)	Density *ρ* (g/cm^3^)	Compressive Strength *σ*_c_ (MPa)	Elastic Compression Modulus *E* (GPa)	Poisson’s Ratio *v*
D-1	50.54	24.62	1.90	22.21	8.29	/
D-2	49.58	24.30	1.90	22.09	5.89	0.46
D-3	50.80	24.32	1.89	19.28	5.89	0.32
D-4	48.40	24.34	1.85	15.82	4.08	0.10
D-5	50.64	24.40	1.87	18.53	6.92	0.05
D-6	49.90	24.34	1.87	18.36	7.76	0.05
Mean value	49.98	24.39	1.88	19.38	6.47	0.20

**Table 4:** Mechanical properties of traditional cement in the literature.

Type	Compressive Strength (MPa)	Elastic Modulus (GPa)
Elastic cement [18]	15.94–31.01	3.19–6.75
High porous cement [21]	15–38	7.95–14.15
Pure cement [32]	18–60	/
Class B cement [38]	25.40–34.79	/
LMCS (Present)	15.82–22.21	4.08–8.29

**Table 5:** Triaxial compression test results of cement stone specimens.

Specimen Number	Confining Pressure ***σ***_3_ (MPa)	Length *L* (mm)	Diameter ***D*** (mm)	Density ***ρ*** (g/cm^3^)	Compressive Strength ***σ***_c_ (MPa)	Elastic Compression Modulus ***E*** (GPa)	Poisson’s Ratio ***v***
S-1	2.0	49.36	24.51	1.90	27.38	5.50	0.05
S-2	4.0	51.03	24.26	1.90	34.90	6.46	0.10
S-3	6.0	49.60	24.60	1.89	37.29	4.48	0.11
S-4	8.0	50.72	24.30	1.85	38.85	6.01	0.16
S-5	10.0	50.18	24.54	1.87	39.58	6.87	0.11
Mean value	6.0	50.18	24.44	1.88	35.60	5.86	0.11

**Table 6:** Brazilian splitting testing results of cement stone specimens.

Specimen Number	Diameter *D* (mm)	Thickness *H* (mm)	Ultimate Load *P*_max_ (N)	Tensile Strength *σ_t_* (MPa)
V-1	24.28	14.12	1738	3.23
V-2	24.54	14.52	1398	2.50
V-3	24.46	14.06	2011	3.72
V-4	24.42	13.15	1179	2.34
V-5	24.20	13.48	1808	3.53
V-6	24.34	14.32	1481	2.71
Mean value	24.37	13.94	1602	3.01

## Data Availability

The original contributions presented in the study are included in the article; further inquiries can be directed to the corresponding author.
